# A pilot study of disulfiram for individuals with persistent symptoms despite prior antibiotic treatment for Lyme disease

**DOI:** 10.3389/fmed.2025.1549324

**Published:** 2025-04-02

**Authors:** Mara Kuvaldina, Jessica Preston, Denise McClellan, Martina Pavlicova, Thomas H. Brannagan, Brian A. Fallon

**Affiliations:** ^1^Lyme and Tick-Borne Diseases Research Center at Columbia University Irving Medical Center, New York, NY, United States; ^2^Department of Psychiatry, Columbia University Irving Medical Center, New York, NY, United States; ^3^Center for Neuroinflammatory and Somatic Disorders, New York State Psychiatric Institute, New York, NY, United States; ^4^Department of Biostatistics, Mailman School of Public Health, Columbia University Irving Medical Center, New York, NY, United States; ^5^Department of Neurology, Columbia University Irving Medical Center, New York, NY, United States

**Keywords:** disulfiram, post-treatment Lyme disease, Chronic Lyme Disease, fatigue, quality of life, clinical trial

## Abstract

**Introduction:**

*In vitro* studies report that disulfiram is effective in killing *Borrelia burgdorferi*. Case series suggest disulfiram may help to reduce the symptoms of patients with persistent symptoms despite prior antibiotic treatment for Lyme disease. This pilot study assessed safety, tolerability, and signs of clinical response.

**Materials and methods:**

Participants with a history of previously treated Lyme disease and persistent fatigue were randomly assigned in a double-blinded fashion to either Group A (disulfiram for 4 weeks and placebo for 4 weeks) or Group B (disulfiram for 8 weeks). Primary outcome endpoint was at 10 weeks with a follow-up at 14 weeks. The primary aim was to assess safety and tolerability. A clinical aim assessed signs of clinical improvement using well-validated measures, focusing on improvement in fatigue and quality of life. Target enrollment was 24 participants.

**Results:**

940 individuals were screened, 11 were enrolled and nine participated in the trial. Dosing started low and increased based on response and tolerance to a maximum of 500 mg daily. Safety. Two participants discontinued medication due to clinical worsening, one of whom was briefly hospitalized. Three additional participants were withdrawn from treatment due to lab test abnormalities. Tolerability. Only three of nine participants completed the full course of treatment (two in Group A and one in Group B). Lower doses were better tolerated than the highest dose. Clinical response. Of nine participants, clinically meaningful improvement was noted in fatigue for six and in quality of life for four. Among the six fatigue responders, improvement was also noted on a multiple domain symptom index (six of six), overall symptom burden (five of six), and functional impairment (four of six). The study was terminated early due to end of project funding, higher than expected adverse events, and recognition that sufficient information was gathered to inform future studies.

**Conclusions and relevance:**

This study reveals the risks associated with disulfiram, especially at higher doses, while suggesting potential clinical benefits among some participants. Efficacy could not be assessed given the small sample size and the lack of a placebo-control group.

**Clinical trial registration:**

https://clinicaltrials.gov/study/NCT03891667?cond=Lyme%20Disease&intr=disulfiram&rank=1, NCT03891667.

## Introduction

The Centers for Disease Control and Prevention (CDC) estimated that 476,000 Americans are diagnosed with Lyme disease (LD) annually based on insurance claims data from 2010 to 2018, making it the most common vector-borne disease in the United States (1). LD is caused by infection with *Borrelia burgdorferi* (*Bb*) and rarely, *Borrelia mayonii*, both of which are transmitted to humans through the bite of an *Ixodes* tick. Untreated LD may lead to disseminated rheumatological, cardiac, and neurological symptoms (2). Although most people recover fully if diagnosed early and treated with antibiotics, a subset of patients may experience persistent or relapsing/remitting symptoms such as pain, fatigue, and neurocognitive problems. Should these symptoms last for more than 6 months after standard antibiotic therapy, the nomenclature for this condition most commonly used in research publications is Post-Treatment Lyme Disease Syndrome [PTLDS; (3, 4)]. Other terms that are less well defined include Long Lyme, Persistent Lyme disease, or Chronic Lyme Disease.

The detrimental impact of PTLDS on functioning and quality of life in affected patients is well-documented (5, 6). Emerging evidence indicates there are many mechanisms that may account for persistent symptoms, including a dysregulated immune response (persistent inflammation, autoimmunity), persistent *Bb* infection or *Bb* remnants, altered microbiome, and/or altered neural circuitry (7, 8). In the United States, two large randomized placebo-controlled antibiotic trials of repeated antibiotic therapy revealed negative findings (9), while a third found sustained benefit on the primary outcome measure of fatigue (10) and a fourth study reported sustained improvement on the secondary outcome measures of pain and physical functioning but not on the primary outcome measure of cognition (11, 12). Each of these studies included intravenous ceftriaxone in the treatment regimen; in each of these studies, the cost-benefit of this treatment was a concern given the risks associated with intravenous ceftriaxone.

The persistent infection hypothesis for continued symptoms gained increased credibility when numerous animal model studies revealed that *B. burgdorferi* can persist despite antibiotic treatment; these persister organisms have been described as viable but not cultivable (13–15). *In vitro* studies demonstrate that these persister forms are less vulnerable to killing with standard antibiotics, presumably because they are in a phase of decelerated growth (16). Rare studies in humans have also demonstrated persistence of the Borrelia microbe after antibiotic treatment (17, 18).

Recent research has focused on identifying pharmacotherapies that can effectively kill both the actively replicating and persister forms (16, 19). A screening study of over 4,000 FDA-approved compounds identified disulfiram as a highly effective bactericidal compound against stationary-phase *B. burgdorferi sensu stricto (Bbss)*, with 99.8% inhibition of metabolic activity at a dose equivalent to 0.38 μg/mL (20). A subsequent mouse study found that treatment with 75 mg/kg disulfiram daily for 5 days led to the reduction or clearance of Borrelia from most tissues (21); however, the very high dose used in this mouse study would be toxic to humans. Other *in vitro* studies have not reported such favorable results for disulfiram. When tested in a stationary-phase *Bbss* culture enriched with persister *Bbss* persister forms, disulfiram was less effective than other antibiotics, such as clarithromycin and nitroxoline (22). In a mouse study of *Bbss* infection, combination therapies that included disulfiram failed to eradicate the *Bbss* organisms, while other combination therapies were effective in eradicating *Bbss* (23).

The possibility that disulfiram, an oral FDA approved drug for moderate to severe alcohol abuse disorder, could be repurposed as a treatment for people with persistent symptoms from Lyme disease caught the attention of patients who then started to ask their physicians to prescribe this medication off-label. The initial report from a clinician's practice of 3 cases (24) was followed by a larger clinical series of 67 patients from the same practice (25); these reports suggested that disulfiram may be helpful for meaningful reduction in persistent symptoms associated with Lyme disease. Remission of symptoms for >6 months without any additional antibiotic therapy was reported by 12 of the 67 patients (17.9%). In that clinical series, the treatment duration ranged from 6 weeks to >16 months. Analysis of the larger series suggested that individuals treated with higher doses were more likely to experience enduring benefit than those treated at lower doses; this higher threshold however was also associated with more adverse events such as fatigue, psychiatric symptoms, peripheral neuropathy, and elevated liver enzymes. These clinical reports were retrospective and did not use standardized measures to assess symptom reduction or side effects.

After publication of the initial *in vitro* disulfiram study (18) and the human report indicating favorable outcome after treatment of three patients (22), we designed the following small prospective pilot study. This double-blind randomized study of 4 vs. 8 weeks of treatment had two primary aims. First, we assessed the safety and tolerability of disulfiram among participants with persistent symptoms despite prior antibiotic treatment for Lyme disease. Second, we investigated whether there was a signal using standardized validated assessments to suggest that treatment with disulfiram results in meaningful reduction in fatigue and improvement in quality of life among participants with persistent symptoms. We hypothesized that disulfiram would be safe and well-tolerated. We further hypothesized that this pilot study data would suggest that disulfiram leads to a reduction in fatigue and an improvement in quality of life.

## Materials and methods

### Ethical approval and registration

This study was approved by the New York State Psychiatric Institute Institutional Review Board (#7755) and registered at ClinicalTrials.gov (“Disulfiram: A Test of Symptom Reduction Among Patients with Previously Treated Lyme Disease,” NCT03891667). All participants signed informed consent.

### Participants

Our target sample size was 24 participants with persistent symptoms despite prior antibiotic treatment for Lyme disease. A rigorous screening process determined potential eligibility. The main inclusion criteria were: (a) age 18–65 and English speaking; (b) a history of fatigue that emerged within 6 months after treatment of well-documented definite or probable Lyme disease and that persisted in a continuous or relapsing-remitting fashion; (c) current fatigue of at least moderate severity, which impairs quality of life, and is not better explained by another condition; (d) a history of at least 5 weeks of treatment using antibiotics typically recommended for Lyme disease (26); (e) a history of at least a partial response to prior antibiotic therapy; (f) the triggering episode of Lyme disease occurred within the prior 16 years during which there was never a symptom-free interval of more than 8 months. The exclusion criteria were extensive given the many contraindications to disulfiram. These included a history of cardiovascular disease, certain psychiatric disorders (including psychosis, bipolar disorder, recent suicidal behaviors, substance abuse or binge drinking), other current tick-borne illness, current renal or liver disease as well as certain neurologic conditions such as a seizure disorder, traumatic brain injury, or large fiber neuropathy. All had to agree to abstain from the use of alcohol and to avoid products that contained alcohol (e.g., cough syrup, sauces, aftershave) for 1 month prior to randomization and for the duration of the study and agree to avoid medication contraindicated with disulfiram. This was done to avoid an adverse alcohol- or medication-related interaction with disulfiram.

### Measures

This study researched the safety and side effect profile of disulfiram among participants with persistent symptoms despite prior antibiotic treatment for Lyme disease. Safety of the treatment was assessed systematically using the SAFTEE at baseline, week 4 and week 10 (27). For this study, a treatment emergent adverse event (TEAE) was considered a symptom that had substantial change from the baseline rating, such as none to moderate severity, or mild to severe severity or any new onset rating of severe. Given the challenges of determining whether a side effect was related to the study drug or due to normal fluctuations in an individual's overall disease, all symptoms with substantial changes in severity rating were recorded as a TEAE, without consideration of relationship to disulfiram. The clinician however did evaluate whether there was a definite/probable, possible/remote, or no relationship to disulfiram.

Neuropathic symptoms were assessed with two measures. The Neuropathy Total Symptom Score-6 (NTSS-6) which evaluates sensory symptoms (28) and the Total neuropathy score (TNSc) which was designed to quantify chemotherapy induced peripheral neuropathy (29). The NTSS-6 measures the frequency and intensity of numbness, allodynia, prickling, and three types of pain (aching, burning and lancinating). The TNSc assesses sensory, motor and autonomic functions, pinprick, vibration, strength, and deep tendon reflexes each being scored 0–4. The total score ranges from 0 to 28; the score represents peripheral neuropathy severity levels of mild (scores of 1–9), moderate (score of 10–19), or severe (scores of 20–28) (30).

For the primary effects of treatment, self-reported measures of fatigue and quality of life were used. Fatigue was assessed by the Fatigue Severity Scale [FSS; (31)] and quality of life was assessed with the Quality-of-Life Enjoyment and Satisfaction Questionnaire [Q-LES-Q; (32)]. For the FSS, a minimal clinically important difference (MCID) is considered a decrease in the total mean score of 0.7 or more (10). Meaningful improvement on the Q-LES-Q is a change score of at least 6.8 points (33).

Secondary outcomes included the PROMIS-29 symptom summary average for the five domains of sleep disturbance, pain, anxiety, depression, and low energy/fatigue [SPADE; (34)], the Short Form-36 (SF-36) Health Survey component summary scores (35), the General Symptom Questionnaire [GSQ-30; (36)], and the Clinical Global Impression—Improvement scale (37, 38).

The PROMIS-29 SPADE score represents the average T-score for the 5 SPADE domains with higher scores indicating greater symptom severity; MCID is considered a reduction in the T-score of at least 3 points (34). The SF-36 is a 36-item, patient-reported survey from which two summary scores can be calculated: Mental Component Summary (MCS; representing mental functioning) and Physical Component Summary [representing physical functioning; (35)]. The MCID chosen for this study was 6.5 for PCS and 7.9 for MCS as described elsewhere (9). The GSQ-30 is a 30 item self-report measure of symptom burden developed for the study of multi-system symptoms associated with infectious illness, such as Lyme disease (36). The measure asks participants to rate how bothered they have been with a particular symptom over a 2-week time frame. Responses are made on a 5-point Likert scale ranging from “not at all” to “very much” (scored 0–4); and the total score ranges from 0 to 120 with higher scores indicating increased symptom severity (36). To assess MCID, we used a cutoff of 50% reduction in the GSQ-30 total score as this is a common cutoff to assess responder status in other symptom-based measures (39, 40). The CGI-I is a clinician-rated scale that assesses the extent of global clinical change at the point of assessment compared with baseline; the score has a 7-point range, from 1 = Very much improved to 7 = Very much worse. A “responder” is an individual with a rating of “much improved” or “very much improved.”

All self-report measures were administered every 2 weeks to the end of treatment at week 8 and again at weeks 10 and 14. The primary outcome timepoint was week 10. Durability of response was assessed at week 14.

### Treatment

Participants were randomized in a 1:1 distribution into one of two treatment arms. Group A was randomized to receive 4 weeks of disulfiram followed by 4 weeks of placebo, while Group B was randomized to receive 8 weeks of disulfiram. The pills were masked such that participants did not know whether they were receiving disulfiram or placebo during weeks 5–8. The dose escalation schedule was individualized for each participant based on preference and side effects using the general guidance of an initial starting dose of 250 mg daily or every other day with an increase over the course of 4 weeks if tolerated to a maximum daily dose of 500 mg daily. During weeks 5–8 the dosage was decreased or increased as needed, but never exceeded 500 mg/daily.

### Procedural changes due to the COVID-19 pandemic

In March 2020 the study was paused due to the COVID-19 pandemic. To enable the study to continue, a fully remote version was launched in 2021. When the pause on in-person research visits was lifted later in 2021, a hybrid version of the study was offered. Participants seen remotely were evaluated by a clinician via telehealth sessions using the same schedule as the initial fully in-person protocol: every 2 weeks to week 10 and then at week 14. In addition, each participant had a weekly contact with a study researcher. As a result of the pandemic, the neuropathy assessments could not be administered to all participants.

### Statistical analysis

Missing data in this study could be only partially treated as missed at random. The COVID-19 disruption and further change of study design led to data missed for a specific reason. We provide only descriptive statistics for the primary clinical outcome assessments for such data. Safety and treatment side-effects were assessed as frequencies of events. Clinical outcomes were assessed via calculation of the number of participants who reached a minimal clinically important difference. For participants who withdrew early, we used the last observation carried forward to assess whether the change between baseline and last assessment reached a magnitude threshold to be considered a minimal clinically important difference. Due to the small number of subjects in the study, we decided to forgo any statistical analyses to avoid type I error and overinterpretation of the results and instead we opted for clear description of the participants and their unique outcomes.

## Results

### Sample description

**Figure 1:**
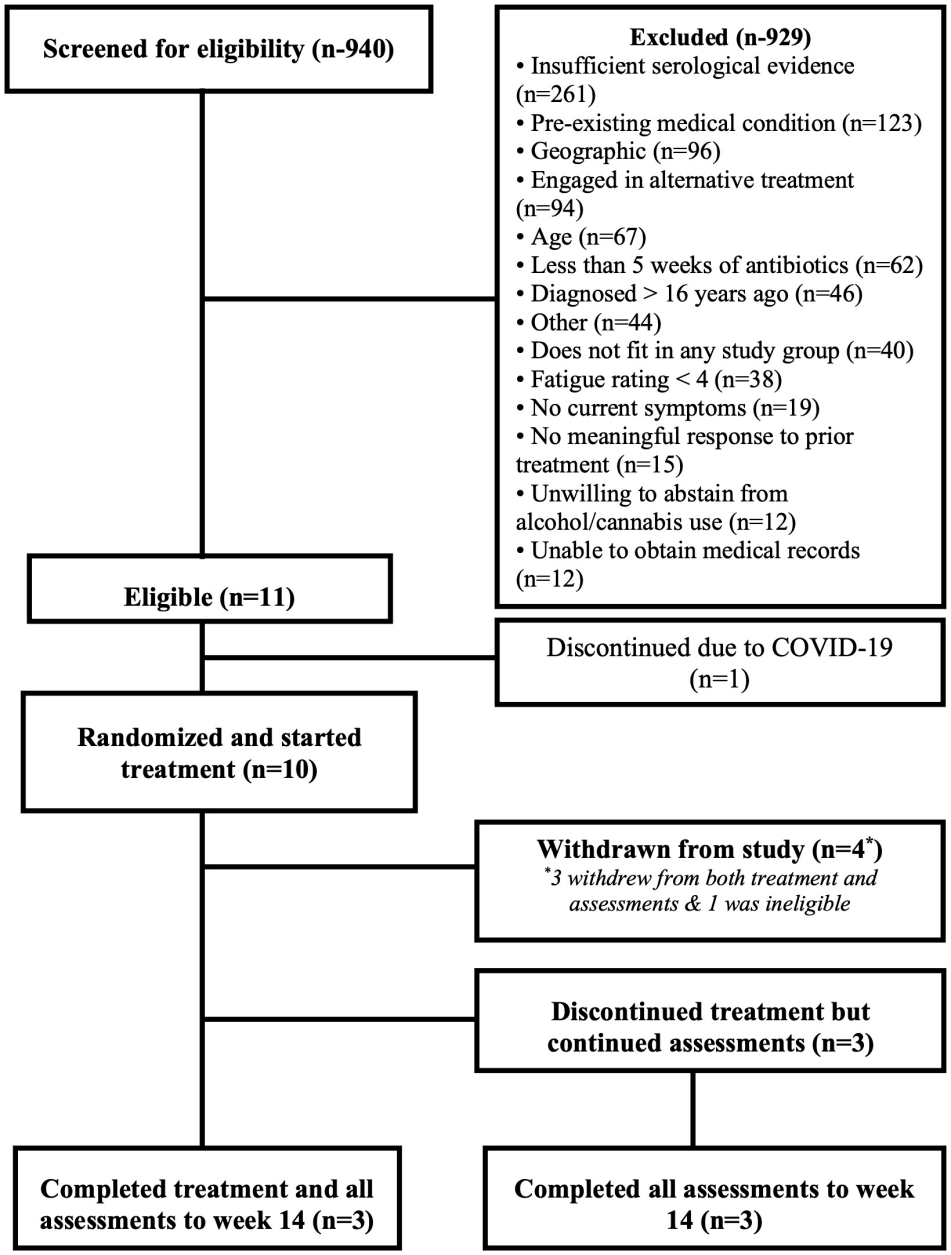
Flowchart of participation in the study.

As indicated in Figure 1, 940 participants were screened for eligibility with more than 900 being ineligible for multiple reasons. The top three obstacles to enrollment were: insufficient serological evidence (27.8%), pre-existing medical conditions (13.1%), and geographic location that prevented visits to clinic (10.2%).

Eleven participants were deemed eligible of whom 10 were randomized and started treatment (five males, five females). Data for one of the randomized participants was removed after an exclusionary pre-existing neurologic condition was identified. See Figure 1. Demographics and baseline scores on self-report measures are presented in Table 1.

**Table 1:** Participants demographics and baseline self-report assessments.

	**Group A (*n* = 4)**	**Group B (*n* = 5)**	**Total (*n* = 9)**
Gender	3 Female (75%)	2 Female (40%)	5 Female (50%)
Age	32.5 (14.4)	38.6 (8.7)	35.9 (10.9)
FSS	5.4 (0.75)	5.7 (0.91)	5.5 (0.8)
Q-LES-Q	43.5 (8.5)	47.4 (5.6)	45.7 (6.9)
SPADE	52.6 (4.6)	55.6 (5.5)	54.2 (5.1)
SF-36 PCS	30.7 (10.8)	38.1 (6.7)	34.8 (9.0)
SF-36 MCS	48.7 (6.5)	41.7 (14.4)	44.8 (11.5)
GSQ-30	36.5(14.3)	41.4 (7.5)	39.2 (10.6)

### Adherence to the study assessments and treatment

Five of the 9 participants (one from Group A and four from Group B) discontinued medication due to adverse reactions: three (Group B) due to elevated liver function tests (of whom one had clinical worsening), one due to an unrelated laboratory abnormality that needed further evaluation (Group B), and one (Group A) due to a possible disulfiram-related reaction. Of these five who discontinued medication, two withdrew from the study completely (medication and assessments) and three continued in the study with assessments to week 14. An additional participant (Group A) did not want to risk being assigned to placebo after week 4 and so withdrew from the study treatment and assessments to be able to continue with privately obtained disulfiram in the community setting. Despite these medication treatment withdrawals at different weeks of the study, six of the nine participants continued with study assessments to week 14. Of the three participants who completed the study medication and assessments fully as per protocol, two were from Group A and one was from Group B.

### Safety and side-effects

There were two serious adverse events (SAE), one of which was likely related to disulfiram use. This individual (Group B) had a 3-day hospitalization due to severe abdominal pain with elevated liver function tests (on 500 mg/day); both pain and abnormal lab tests resolved with discontinuation of disulfiram. The second individual (Group A) was treated in the emergency room for a possible emerging anaphylactic reaction; as this occurred more than 10 days after discontinuation of disulfiram, it was deemed unlikely to be disulfiram related. Both participants recovered fully from these adverse events.

Safety of the treatment and side-effects were assessed systematically using the SAFTEE tool at each major study visit (at baseline, week 4 and week 10). Of the nine participants, six reported at least one treatment emergent adverse event (TEAE). In Group A, one of four participants experienced two TEAE (temperature fluctuations and shooting pain). In Group B, all five participants reported at least one TEAE. Of these five participants, one person experienced six TEAE (dizziness, photosensitivity, neuromotor weakness, throat hoarseness, tinnitus, sleepiness), one experienced three TEAE (headache, sore throat, mild elevation of serum creatinine) and the other three participants experienced one TEAE each (elevated liver function in two participants and sleepiness in one participant). Among these non-serious clinical TEAEs, only three were rated by the clinician to be probably associated with disulfiram treatment (headaches, sleepiness, throat hoarseness). Including the individual with a SAE, elevated liver enzymes were noted in three participants from Group B; two had reached the maximum dose of 500 mg/daily (with disulfiram discontinuation after 3 weeks and 6 weeks) and one had reached a maximum dose of 250 mg/daily (with disulfiram discontinuation after 6 weeks). The liver enzyme elevation was not associated with clinical worsening suggestive of liver toxicity in two of the three cases. None of the four individuals in Group A had elevated liver enzymes and none had reached the maximum dose (500 mg/daily) of disulfiram. Two of the three individuals who reached the maximum dose of 500 mg daily were withdrawn from study treatment due to elevated liver enzymes. The personalized dosing schedule of disulfiram was personalized for participants in this study, ranging from a maximum of 250 mg every other day to 500 mg/day.

Potential neurotoxicity of disulfiram was assessed by a clinician by comparing ratings on the TNSc and NTSS-6 scales before and after completing 8 weeks of the treatment study. Among the six participants with assessments at both time points, none developed a neuropathy or significant worsening of pre-treatment neuropathic symptoms. Two of the 6 reported neuropathic symptoms before treatment that were unchanged after treatment.

## Primary clinical self-report assessments

Another goal of the study was to assess whether there are signals in the data suggesting that disulfiram leads to improvement in some of the core symptoms of post-treatment Lyme disease. The two primary clinical outcome measures were fatigue as measured by Fatigue Severity Scale (31) and Quality of Life satisfaction as assessed by the Q-LES-Q (32). See Table 2.

**Table 2:** Clinical response of participants.

**Participant**	**Group**	**FSS**	**Q-LES-Q**	**SPADE**	**SF-36 PCS**	**SF-36 MCS**	**GSQ-30 change from baseline**	**Final dose of disulfiram**	**Last week of disulfiram treatment**	**Last** **assessment**
**1**	A	NR	NR	NR	–	–	−27.1%	250 mg/day	Week 2	Week 3
**2**	A	NR	NR	NR	NR	NR	−50%	250 mg every other day	Week 4	Week 5
**3**	A	**R**	**R**	**R**	**R**	**R**	**57.1%**	250 mg every other day	Week 4	Week 14
**4**	A	**R**	**R**	**R**	**R**	NR	**68.9%**	250 mg/day	Week 4	Week 14
**5**	B	**R**	NR	**R**	NR	NR	48.8%	500 mg/day	Week 6	Week 14
**6**	B	**R**	NR	**R**	NR	NR	**70.7%**	250 mg/day alternating with 500 mg/day	Week 8	Week 14
**7**	B	**R**	**R**	**R**	NR	**R**	**76.9%**	500 mg/day	Week 3	Week 14
**8**	B	**R**	**R**	**R**	**R**	**R**	**77.5%**	250 mg/day	Week 6	Week 14
**9**	B	NR	NR	NR	–	–	22.5%	250 mg/day	Week 3	Week 3

Group A randomized to disulfiram (4 weeks) and placebo (4 weeks). Group B randomized to disulfiram (8 weeks).

FSS, Fatigue Severity; Q-LES-Q, Quality of Life; SPADE, Multi-domain Symptom Severity Index; SF-36 PCS, index of physical functioning; SF-36 MCS, index of mental functioning; GSQ-30, Multi-system Symptom Burden.

Change scores from baseline to last assessment that are of sufficient magnitude to be considered clinically meaningful (i.e., a responder on the measure) are highlighted in bold. R, Responder; NR, non-Responder.

### Fatigue

Six of nine participants treated with disulfiram reached or exceeded the MCID indicating clinically significant improvement in fatigue: two of four in Group A and four of five in Group B. The improvement among these six individuals was robust, as responder status was also noted on other measures: multi-domain illness severity (SPADE; six of six), overall symptom burden (five of six), quality of life (four of six), and either physical or mental functioning (four of six). Among the responders on the Fatigue Severity Scale, the highest dose during the treatment was 500 mg/day for three participants, 250 mg/day for two participants, and 250 every other day for one participant.

### Quality of life improvement

On the Q-LES-Q, four of nine participants treated with disulfiram had scores indicating a meaningful improvement in their quality of life: two of four in Group A and two of five in Group B.

## Secondary clinical outcome assessments

### Multi-domain illness severity

For meaningful change on this illness index (SPADE from PROMIS-29), six of nine were improved: two of four in Group A and four of five in Group B.

### Physical or mental health status

For meaningful change in physical function (SF-36 PCS), three of nine were improved: two of four in Group A and one of five in Group B. For meaningful change in mental health functioning (SF-36 MCS), three of nine were improved: one of four in Group A and two of five in Group B.

### Symptom burden

For meaningful change in symptom burden assessed with GSQ-30, five of nine were improved: two in Group A and three in Group B.

### Clinician-rated clinical global improvement

Using the CGI-I, the clinician compared overall clinical outcome compared to baseline status. At the primary assessment endpoint after treatment, five of six participants received ratings consistent with responder status: two in Group A and three in Group B.

### Durability to week 14

Five of six participants rated as fatigue responders continued to show clinically meaningful improvement in fatigue at week 14. On the Q-LES-Q, three of the four participants rated as responders at end of treatment continued to show clinical improvement in quality of life to week 14. On the clinician rating of global improvement, five individuals continued to be rated as responders at week 14.

## Discussion

This double-blind placebo-controlled randomized study was designed to assess safety, tolerability, and initial signs of effectiveness of disulfiram in reducing symptoms among patients with persistent symptoms despite prior antibiotic treatment for Lyme disease. While our goal was to enroll 24 participants, we stopped enrollment after nine participants completed the study. The study was stopped due to delays in enrollment related to the COVID pandemic, end of project funding, higher than expected adverse events, and recognition that sufficient information was gathered to inform future studies. Due to the small sample size, the results reported in this paper must be recognized as preliminary, qualitative, and meaningful only in so far as they provide guidance for future studies.

The extensive literature on disulfiram indicates that the most common side effects include drowsiness, fatigue, headache, papular acne, sexual dysfunction, and a metallic taste. Rarely, serious life-threatening risks have been reported (41). National guidelines recommend disulfiram as a second line treatment for moderate to severe alcohol abuse disorder with the stipulation that disulfiram should only be given to those who are capable of understanding the serious risks associated with the interaction of alcohol and disulfiram and to those who have no contraindications to the use of this medication (42). Exposure to alcohol in any form can lead to the disulfiram ethanol reaction (DER) which can range from mild symptoms to life-threatening adverse events (41, 42). While avoidance of alcoholic beverages is essential for individuals on disulfiram, other inadvertent exposures may also put the patient at risk for DER. Examples of such exposures include the use of alcohol containing products, such as certain hand sanitizers, personal hygiene items, and cooking products; the ethanol may be absorbed through skin contact or inhalation. Certain elixir formulations of common over-the-counter or prescribed medications (e.g., acetaminophen, diphenhydramine, sertraline) may contain sufficient ethanol to trigger a DER (43). Before disulfiram is prescribed, drug interactions need to be checked. Because disulfiram inhibits cytochrome P450 reductase in the liver, the metabolism of other medications that use this pathway will be inhibited, potentially leading to toxic levels (e.g., warfarin and phenytoin). Rarely, disulfiram may induce serious adverse events even without alcohol exposure such as liver failure, cardiac toxicity, psychosis, and peripheral and central neurotoxicity (41). A thorough risk-benefit discussion needs to be conducted with the patient whenever disulfiram is prescribed.

To optimize safety in our study we excluded individuals who might be at higher risk of adverse events due to prior medical or psychiatric history. Therefore, the safety profile from this pilot study of individuals with a history of Lyme disease may not be generalizable to the much larger population, many of whom will have other medical or psychiatric comorbidities. Elevated liver function tests leading to medication discontinuation were noted in three of the nine study participants; this finding is consistent with published studies in other non-Lyme populations (44, 45). Liver function tests need to be regularly assessed as levels can rise quickly, particularly at doses above 250 mg daily. In our study we checked liver function tests every 2 weeks; when elevated levels were noted, disulfiram was discontinued, and the levels returned to normal range. Of the two serious adverse events, the one deemed likely related to disulfiram may have been due to either inadvertent alcohol exposure or the toxic action of disulfiram or its metabolites on the liver in the absence of concurrent alcohol exposure (45). None of our participants developed neuropathic symptoms during the study. Regarding safety, we conclude that disulfiram is a medication that requires careful monitoring due to the potential for serious risks.

Regarding tolerability, while six of the nine participants continued assessments to the end of the study, only three of nine participants stayed on study treatment for the full 8 weeks of the study. The most common reason for treatment withdrawal was elevation of the liver function tests. While Group B members were more likely to report a worsening of symptoms or experience abnormal lab tests compared to Group A participants, the numbers in each group are too small to draw conclusions about whether longer vs. shorter duration treatment is preferable. These clinical and laboratory adverse events suggest that disulfiram is not well tolerated; while higher disease doses of disulfiram are associated with increased risk, even the lower dose of 250 mg/day may lead to elevated liver enzymes as noted in one participant in our study. As noted in a prior disulfiram clinical series (25), it is likely that adverse events would have been less if the dosing started lower, increased more slowly, and the maximum dose was kept as low as possible.

Our study suggests that disulfiram may lead to a meaningful reduction in symptoms, as six of the nine participants reported benefit from the study medication on the primary outcome of fatigue. This is encouraging as the chronic symptoms associated with Lyme disease can be quite debilitating. However, these results must be interpreted cautiously. There has been considerable enthusiasm on social media about disulfiram as a treatment for persistent Lyme disease associated symptoms, leading to a bias toward a strong expectation of benefit. Without a placebo-control group, we cannot say whether the improvement among these individuals was due to disulfiram or not. We can say that the improvement among these responders was seen in multiple domains, both on self-report and clinician ratings. We can also say that in general the improvement had short-term durability (lasting at least 6 weeks after finishing study treatment).

A prior clinical series suggested that higher doses and longer duration of disulfiram treatment are associated with an increased likelihood of benefit (24, 25). In our small study, five of the six fatigue responders had had no more than 6 weeks of disulfiram and responders were seen at both lower and higher doses of disulfiram. This is a hopeful finding and suggests that a future placebo-controlled study should consider shorter durations of treatment with doses at < 500 mg/day to reduce the risk of adverse events (41).

Strengths of this study include the use of standardized measures to assess clinical response and side effects, masking of pills to ensure blinding about randomization to Group A or B, flexibility in dosing to individual tolerance and response, blood test monitoring every 2 weeks, and employment of a fully remote version of the protocol during the COVID pandemic. From a design perspective, our study suggests that a fully remote or hybrid study design (part in person and part remote) works well for participants and should be considered for future clinical research studies The main limitation is the small sample size which prevents a meaningful comparison of short vs. longer term treatment; the lack of a placebo control group also prevents an assessment of efficacy. Additional limitations include not being able to assess whether participants who responded to treatment had an enduring response beyond the 6 week follow-up in our study and, in our analysis, not being able to evaluate response and tolerability by patient dose and body weight, as has been done in a prior publication (25).

Notable in our study is that only 1% of those screened were enrolled and randomized; this limits the generalizability of our study results. Because our study aimed to assess the impact of disulfiram as a potential antibiotic, we were careful to ensure that all participants had sufficient evidence to confirm the prior diagnosis of Lyme disease. Such diagnostic rigor is needed, particularly when testing an antimicrobial treatment. Future studies addressing other treatment approaches for persistent symptoms should consider enrolling participants with a credible history of Lyme disease (even if definitive confirmatory documentation is not available), thereby increasing recruitment rate and study feasibility and enhancing generalizability to the wider diversity of patients with persistent or relapsing Lyme disease-associated symptoms commonly seen in clinical practice (46, 47). To enhance the interpretation of such studies, participants should be classified prospectively based on diagnostic certainty to enable subgroup analyses.

Researchers have continued to seek other antibiotic regimens that may be effective in killing both actively replicating and persister Borrelia (48). Given the observation that other microbes associated with persistence, such as *Mycobacterium tuberculosis*, require combination therapy for effective treatment, this approach has been explored for eradication of *B. burgdorferi*. *In vitro* studies suggest combination therapy leads to improved efficacy against *B. burgdorferi* (20, 48). Recent mouse studies (23) demonstrated that of the evaluated monotherapies (including disulfiram), none were able to eradicate persistent Bb. However, four of the dual combinations and three of the triple combinations were effective in eradicating persistent Bb infections. These studies provide new directions for investigation. Further human research is needed to identify well-tolerated and effective treatment options for patients with persistent symptoms. Researchers should also explore non-antimicrobial therapeutic approaches as mechanisms of disease other than persistent infection can play a prominent role in persistent symptoms, such as immune or neural dysregulation or dysbiosis (7, 49).

In summary, the main finding was that disulfiram treatment was not well-tolerated at the dosing schedule used for this small pilot study. Although clinical benefit was reported by more than half of the participants, meaningful conclusions cannot be drawn about the potential benefit of disulfiram for patients with persistent symptoms after Lyme disease as the sample size was small and the study was not placebo-controlled.

## Data Availability

The raw data supporting the conclusions of this article will be made available by the authors, without undue reservation.
